# The global epidemiology of gastrointestinal cancer burden attributable to dietary risks: a systematic analysis of the Global Burden of Disease Study 2021

**DOI:** 10.3389/fnut.2025.1677735

**Published:** 2025-10-23

**Authors:** Laiang Yao, Jiao Nie, Lingjian Kong, Shuai Shao, Xiangming Xu

**Affiliations:** Department of Gastroenterology, Linyi People’s Hospital, Linyi, Shandong, China

**Keywords:** gastrointestinal cancers, colorectal cancer, dietary risk factors, global burden of disease, nutrition and cancer, gastroenterology

## Abstract

**Background:**

Gastrointestinal (GI) cancers collectively account for over 30% of global cancer-related mortality, with diet and nutrition playing crucial roles in their development. We investigated the burden, trends and disparities of GI cancers attributable to dietary risks.

**Methods:**

Data was collected from the Global Burden of Disease Study 2021. Disease burden was measured by deaths and disability-adjusted life years (DALYs), along with age-standardized rates (ASRs). Joinpoint regression, with average annual percent changes (AAPCs) were used to assess temporal trends. ARIMA models were employed to project the ASRs till 2040.

**Results:**

Between 1990 and 2021, the AAPC of the age-standardized mortality rate (ASMR) and age-standardized DALY rate (ASDR) of colorectal cancer (CRC) attributable to dietary risks were −0.87 (95% CI: −0.89, −0.84) and −0.88 (95% CI: −0.90, −0.86). Esophageal cancer showed the greatest declining rate, with ASRs declining more than 3% annually. The ASRs of stomach cancer decreased by more than 2% per year. The burden of stomach cancer and esophageal cancer were highest among low and low-middle SDI countries and regions, particularly East Asia and Sub-Saharan Africa, respectively. High-SDI countries and regions showed the highest burden of CRC but the greatest declining rates. Future projections suggest constant decreasing burden for stomach cancer and CRC, but stable trends for esophageal cancer.

**Conclusion:**

Diet-attributed GI cancer remains a significant public health challenge globally, especially among low SDI and lower-middle SDI countries. Given the disparity of risk exposures and disease burden, we recommend promoting screening practices and improving healthcare accessibility in low SDI countries, while emphasizing lifestyle modifications in higher SDI countries to combat this pressing issue.

## Introduction

Gastrointestinal (GI) cancers constitute a range of malignant conditions affecting the digestive system, including gastric, colorectal, liver, esophageal, and pancreatic cancer (1, 2). Recent statistics from the GLOBOCAN database indicate that GI cancers collectively represent approximately one-fourth of all cancer incidences and a third of all cancer-related mortalities (3–5). A recent population-based systematic analysis has revealed that the lifetime risk of developing and dying of GI cancers stands at 8.2 and 6.17%, respectively (6). Moreover, previous projection analyses have highlighted an expected increase in the incidence and mortality burden of various GI cancer subtypes in the future (7, 8).

Despite the substantial and steadily increasing burden of GI cancers on global public health, it is noteworthy that the majority of these cancers are attributable to modifiable, and therefore preventable, risk factors. Evidence from U.S. cancer registries and large-scale pooled cohort studies indicates that over 40% of GI cancer incidence and mortality can be attributable to such modifiable factors (9, 10). Among them, dietary risks represent a particularly critical determinant, as the consumption of specific foods and nutrients may either elevate or reduce cancer risk (11–13). A growing body of evidence has established clear associations between GI cancers and various dietary patterns. For example, a systematic review and meta-analysis demonstrated that consumption of red meat and processed meat increased the risk of colorectal cancer by 10 and 18%, respectively (14). Conversely, a daily intake of three servings of whole grains was associated with a 17% reduction in colorectal cancer risk (15).

While the strong association between GI cancers and diet is well-established, limited research focused on quantifying the disease burden of GI cancers attributable to dietary risks. Previous Global Burden of Disease (GBD) studies have quantified the overall burden of GI cancers, but a detailed, longitudinal analysis focusing specifically on dietary risk factors, such as diets high in red meat and low in fruits, fiber, and whole grains, has been lacking. Consequently, critical knowledge gaps persists regarding the temporal trends and geographical disparities of GI cancers attributable to dietary risks. To address this need, we utilized data from the Global Burden of Disease (GBD) 2021 study, which is the most comprehensive global effort to estimate disease burden and risk factors. Drawing on this dataset, our study systematically investigates the burden, temporal trends, and regional as well as national disparities of GI cancers attributable to dietary risks. We also quantified and assessed the disease burden attributable to specific dietary risk factors (diet high in red meat, diet low in whole grains) to gain a deeper understanding of the nutritional epidemiology. We further projected and forecasted the disease burden till 2040. Ultimately, our studies aim to provide critical evidence to inform public health dietary guidelines and support the development of effective nutritional interventions to reduce the global burden of GI cancers.

## Methods

### Data source

We obtained our data from the Global Burden of Disease Study 2021, which is a comprehensive global health study coordinated by the Institute of Health Metrics and Evaluation (IHME) at the University of Washington. GBD 2021 provides the most up-to-date estimates of over 300 diseases and injuries, along with more than 80 risk factors across 204 countries and territories (16). Data was publicly available through the online query tool Global Health Data Exchange.^1^ Our study followed the Guidelines for Accurate and Transparent Health Estimates Reporting Guidelines for cross-sectional studies (GATHER) (17).

### Definitions

Dietary risks were defined as the average daily consumption of specific food groups and items lower or higher than the optimal level or range of intake. Dietary data was obtained from various sources, including dietary recall, food frequency questionnaires, household budget surveys, food availability data from the Food and Agriculture Organization and so on. The detailed definitions, data sources and estimation process has been previously reported by GBD 2021 collaborators (16, 18). In our study, we used deaths and DALYS (disability-adjusted life years) to measure disease burden. Mortality data was primarily obtained from cancer registries, vital registration systems and verbal autopsy studies. DALYs is a metric that sums the years of life lost (YLLs) with premature mortality and years of life with disability (YLDs), thereby capturing both the fatal and nonfatal components of disease burden. One DALY represents 1 year of loss of optimal health. Various statistical techniques, such as garbage code redistribution and misclassification correction were employed to ensure consistency of the data. Disease burden estimates were performed with Bayesian meta-regression and ensemble modelling strategies including CODEm (Cause of Death Ensemble model) and Dis-Mod-MR 2.1.

A comparative risk assessment method (CRA) was employed to quantify the disease burden of GI cancers attributable to dietary risks, with detailed methodologies provided within previous publications (18). In brief, this method involves four main steps: (1) estimation of the exposure and distribution of each attributable risk factor; (2) calculation of relative risks (RRs) for each risk-outcome pair based on pooled studies and meta-analyses of epidemiological studies; (3) specification of the theoretical minimum risk exposure level (TMREL), which represents the counterfactual level of exposure associated with the lowest population risk, which may correspond to zero intake (e.g., processed meat) or to the optimal range of consumption (e.g., whole grains) and (4) calculation of the population attributable fraction (PAF) based the RR and TMREL, with the formula expressed as below:


$$\mathrm{PAF}=\frac{\sum_{i}P(x)\times (RR_{\left(x\right)}-RR_{\left(x*\right)})}{\sum_{i}P(x)\times \mathrm{RR}(x)}$$

where *P*(*x*) represents the population distribution of exposure, *RR*(*x*) presents the relative risk at exposure level x and *RR*(x*) representing the counterfactual relative risk at the TMREL level. PAF estimates the proportional reduction in disease burden that would be achieved if exposure level was shifted to the TMREL. CRA incorporates a risk mediation matrix to address the non-independence among risk factors, which ensures overlapping effects are partitioned appropriately, thereby avoiding over-counting of attributable burden.

GBD estimated disease burden attributable to risk factors with a hierarchy of four levels, with level 1 being environmental/occupational risks, behavioral risks and metabolic risks while level 4 being the most detailed risk factor. In this study, we used the level 2 risk of dietary risks, which is a composite estimate of the dietary risk exposures, with eight level three detailed risk factors. GBD has identified three GI cancers attributable to dietary risks, which are stomach cancer, esophageal cancer, and colorectal cancer. Stomach cancer and esophageal cancer were attributable to a single dietary risk, which is diet high in sodium and diet low in vegetables, respectively. Colorectal cancer (CRC) was attributable to a total of six dietary risks, including diet low in whole grains, fiber, milk, calcium and diet high in red and processed meat. Socio-demographic index (SDI) indicates the social and economic conditions of a certain region that influences health outcomes. SDI is calculated as the geometric mean of total fertility rate, lag distributed income per capita and mean education level on a scale of 0 to 100. Countries and territories were defined as low, low-middle, middle, high-middle and high SDI according to quintiles of the location-specific SDI values.

### Statistical analysis

We analyzed the disease burden of GI cancers attributable to dietary risks across various demographics, including age, sex, temporal dimensions and geographical locations. The total number of deaths and DALYs, age-standardized rates (ASR), along with the 95% uncertainty interval (95% UI) was reported. ASRs were calculated with adjustments based on the global age structure. The 95% UIs were defined as the 25th and 975th values of the 1000 draw estimates. Joinpoint regression (National Cancer Institute, Rockville, MD, United States) was used to calculate the annual percentage change (APC) and average annual percentage change (AAPC) of the ASRs of GI cancer attributable to dietary risks. An increasing trend was determined with the lower limits of the 95% confidence interval greater than 0 while the upper limit of the 95% confidence intervals less than 0 indicates decreasing trends. Locally estimated scatterplot smoothing (LOWESS) regression models were used to explore the association between SDI with the disease burden of GI cancers attributable to dietary risks across different locations and years. ARIMA (autoregressive integrated moving average) models were used to project and forecast the disease burden till 2040. All data analysis, visualizations were performed with RStudio (v.2024.04.2).

## Results

### The overall impact and the temporal trends

**Figure 1:**
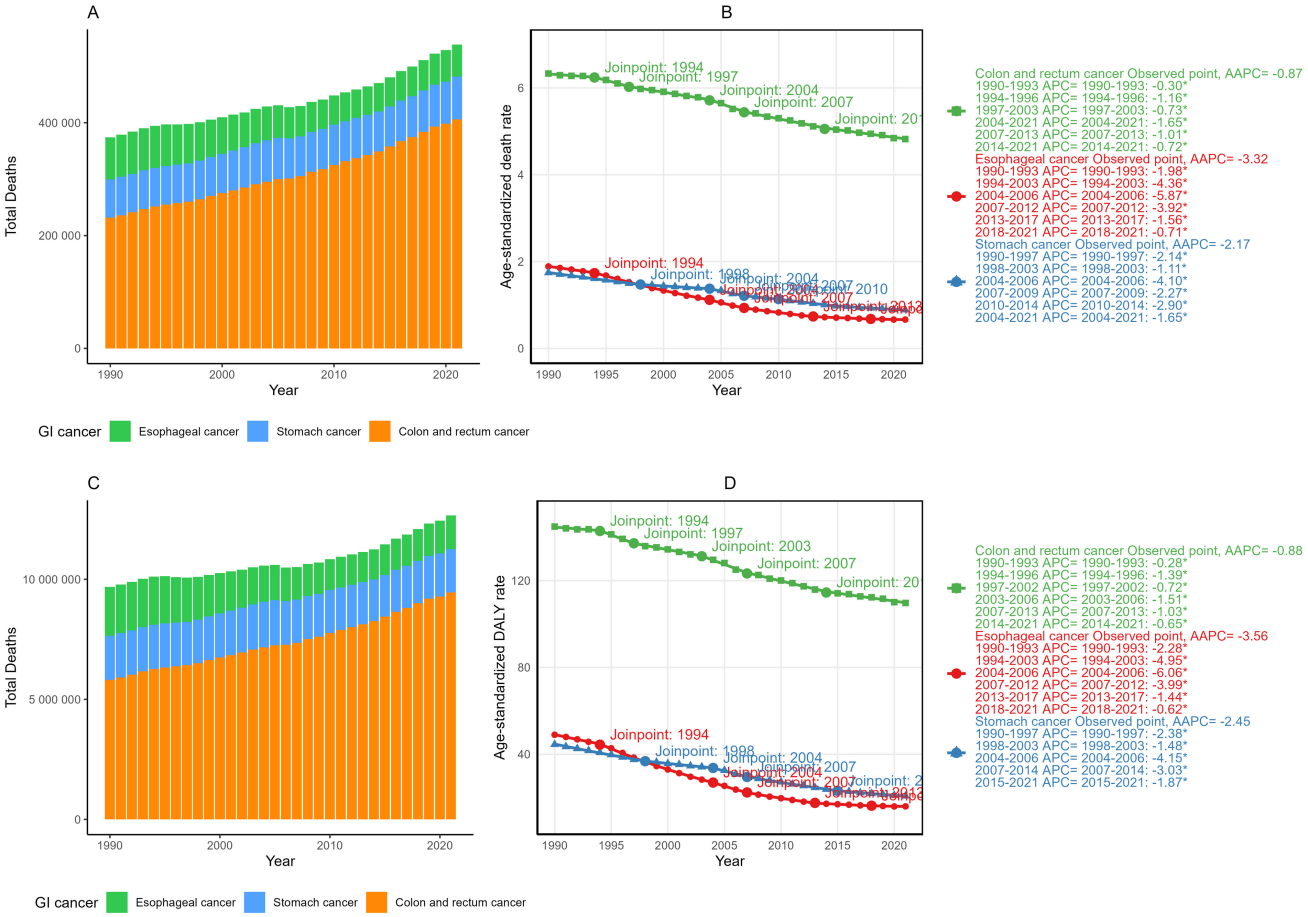
The total deaths **(A)** and DALYs **(C)** of gastrointestinal cancers attributable to dietary risks from 1990 and 2021; The temporal trends, average annual percent changes (AAPCs) of the age-standardized mortality rate (ASMR) **(B)** and age-standardized DALY rate (ASDR) **(D)** of gastrointestinal cancers attributable to dietary risks.

In 2021, dietary risks were estimated to account for approximately 406,000 deaths and 9.46 million DALYs from colorectal cancer (CRC), 56,900 deaths and 1.4 million DALYs from esophageal cancer, and 75,700 deaths and 1.8 million DALYs from stomach cancer (Figure 1 and Tables 1, 2). Between 1990 and 2021, the total deaths and DALYs from CRC nearly doubled (232,000 deaths and 5.81 million DALYs in 1990), while esophageal cancer had significant decreases (74,400 deaths and 2 million DALYs in 1990) and stomach cancer remained relatively stable (67,800 deaths and 1.8 million DALYs in 1990). Globally, age-standardized rates (ASRs) of all gastrointestinal cancers attributable to dietary risks decreased, with esophageal cancer showing the steepest decline and CRC the smallest. By 2021, the global age-standardized mortality rate (ASMR) and age-standardized DALY rate (ASDR) of esophageal cancer was 0.66 and 16.00 per 100,000 population, respectively, with average annual percentage changes (AAPCs) of −3.32% (95% CI: −3.35 to −3.30) and −3.56% (95% CI: −3.58 to −3.53). For CRC, the ASMR and ASDR decreased annually by −0.87% (95% CI: −0.89 to −0.84) and −0.88% (95% CI: −0.90 to −0.86), corresponding to ASRs of 4.82 (95% UI: 1.64–7.46) and 109.7 (95% UI: 37.7–168.5) per 100,000 population in 2021. Stomach cancer showed intermediate declines, with AAPCs of −2.17% (95% CI: −2.19 to −2.15) for ASMR and −2.45% (95% CI: −2.47 to −2.43) for ASDR.

**Table 1:** Deaths, age-standardized mortality rates (ASMR) and average annual percent changes (AAPC) of gastrointestinal cancers attributable to dietary risks in 1990 and 2021*.

	Colon and rectum cancer					Esophageal cancer					Stomach cancer				
	1990		2021			1990		2021			1990		2021		
	Deaths	ASMR	Deaths	ASMR	AAPC of ASMR	Deaths	ASMR	Deaths	ASMR	AAPC of ASMR	Deaths	ASMR	Deaths	ASMR	AAPC of ASMR
Global	231,758 (83,612, 346,321)	6.33 (2.26, 9.47)	406,099 (138,056, 628,056)	4.82 (1.64, 7.46)	−0.87 (−0.89, −0.84)	74,453 (−16,380, 147,090)	1.89 (−0.42, 3.73)	56,939 (−12,023, 118,370)	0.66 (−0.14, 1.38)	−3.32 (−3.35, −3.30)	67,845 (0, 339,513)	1.74 (0, 8.74)	75,661 (0, 372,194)	0.89 (0, 4.37)	−2.17 (−2.19, −2.15)
Sex															
Male	112,991 (37,861, 171,949)	6.95 (2.28, 10.55)	217,748 (68,756, 338,575)	5.74 (1.81, 8.94)	−0.65 (−0.68, −0.62)	51,410 (−11,393, 102,783)	2.82 (−0.63, 5.66)	39,364 (−8,479, 83,291)	1.0 (−0.22, 2.1)	−3.79 (−3.97, −3.61)	43,642 (0, 220,845)	2.46 (0, 12.43)	50,374 (0, 247,168)	1.29 (0, 6.34)	−2.12 (−2.21, −2.02)
Female	118,766 (44,951, 176,650)	5.84 (2.2, 8.69)	188,351 (68,045, 280,577)	4.06 (1.47, 6.04)	−1.30 (−1.34, −1.25)	23,044 (−4,415, 46,696)	1.09 (−0.21, 2.22)	17,575 (−3,679, 36,468)	0.38 (−0.1, 0.79)	−3.86 (−4.09, −3.62)	24,202 (0, 123,378)	1.15 (0, 5.86)	25,287 (0, 129,118)	0.55 (0, 2.79)	−2.57 (−2.67, −2.48)
Region															
Central Asia	1,900 (570, 2,954)	4.09 (1.24, 6.35)	2,375 (652, 3,809)	3.04 (0.85, 4.84)	−0.62 (−0.75, −0.48)	1,046 (−226, 2,165)	2.31 (−0.5, 4.76)	233 (−43, 544)	0.32 (−0.06, 0.74)	−7.52 (−8.02, −7.00)	998 (0, 4,972)	2.13 (0, 10.58)	723 (0, 3,725)	0.9 (0, 4.6)	−2.54 (−2.64, −2.44)
Central Europe	12,131 (3,434, 18,846)	8.33 (2.37, 12.93)	19,615 (5,014, 30,483)	8.55 (2.16, 13.44)	−0.01 (−0.14, 0.11)	782 (−169, 1,603)	0.53 (−0.11, 1.08)	691 (−140, 1,454)	0.32 (−0.06, 0.66)	−2.14 (−2.31, −1.98)	2,259 (0, 11,339)	1.54 (0, 7.69)	1,600 (0, 7,856)	0.71 (0, 3.49)	−2.58 (−2.67, −2.50)
Eastern Europe	21,111 (5,045, 32,306)	7.60 (1.83, 11.63)	25,628 (6,491, 39,262)	7.18 (1.82, 11.01)	−0.45 (−0.59, −0.32)	2,242 (−477, 4,501)	0.8 (−0.17, 1.6)	1,828 (−361, 3,730)	0.52 (−0.1, 1.06)	−1.34 (−1.66, −1.03)	6,323 (0, 32,996)	2.25 (0, 11.72)	3,241 (0, 16,645)	0.92 (0, 4.74)	−3.11 (−3.21, −3.01)
Australasia	2,274 (562, 3,614)	9.8 (2.42, 15.59)	3,300 (800, 5,254)	5.84 (1.4, 9.29)	−1.86 (−1.94, −1.78)	219 (−49, 434)	0.93 (−0.21, 1.84)	429 (−96, 855)	0.77 (−0.17, 1.53)	−0.70 (−0.78, −0.62)	113 (0, 630)	0.49 (0, 2.7)	138 (0, 788)	0.25 (0, 1.41)	−2.10 (−2.22, −1.99)
High-Income Asia Pacific	13,572 (4,010, 20,891)	7.01 (2.1, 10.84)	30,819 (9,926, 47,993)	5.76 (1.76, 8.94)	−0.67 (−0.72, −0.61)	1,489 (−303, 3,087)	0.74 (−0.15, 1.54)	1,767 (−335, 4,025)	0.35 (−0.06, 0.79)	−2.32 (−2.50, −2.14)	6,059 (0, 29,937)	3.09 (0, 15.62)	5,864 (0, 29,532)	1.09 (0, 5.43)	−3.44 (−3.48, −3.39)
High-income North America	29,383 (6,726, 45,806)	8.18 (1.86, 12.74)	34,423 (7,042, 54,451)	5.2 (1.05, 8.22)	−1.58 (−1.64, −1.52)	2,418 (−518, 4,830)	0.69 (−0.15, 1.39)	4,461 (−975, 8,984)	0.67 (−0.15, 1.35)	−0.04 (−0.20, 0.13)	1,414 (0, 7,466)	0.4 (0, 2.1)	1,482 (0, 7,675)	0.23 (0, 1.17)	−1.88 (−1.92, −1.83)
Southern Latin America	3,633 (1,137, 5,561)	8.16 (2.58, 12.46)	6,541 (1,856, 10,210)	7.35 (2.58, 12.46)	−0.07 (−0.22, 0.08)	779 (−180, 1,542)	1.73 (−0.4, 3.42)	764 (−178, 1,537)	0.86 (−0.2, 1.72)	−2.23 (−2.42, −2.03)	686 (0, 3,455)	1.52 (0, 7.61)	752 (0, 3,767)	0.85 (0, 4.26)	−1.69 (−1.80, −1.58)
Western Europe	54,456 (12,646, 84,848)	9.18 (2.12, 14.31)	62,171 (13,416, 98,755)	6.01 (1.27, 9.52)	−1.41 (−1.45, −1.37)	5,140 (−1,127, 10,310)	0.9 (−0.2, 1.8)	6,828 (−1,508, 13,840)	0.72 (−0.16, 1.45)	−0.73 (−0.81, −0.65)	6,132 (0, 32,292)	1.04 (0, 5.47)	4,050 (0, 21,331)	0.41 (0, 2.1)	−3.00 (−3.10, −2.90)
Andean Latin America	721 (358, 1,044)	3.74 (1.87, 5.42)	2,224 (888, 3,420)	3.85 (1.54, 5.91)	0.12 (0.02, 0.22)	102 (−22, 201)	0.53 (−0.12, 1.05)	206 (−47, 405)	0.36 (−0.08, 0.71)	−1.33 (−1.44, −1.22)	522 (0, 2,645)	2.67 (0, 13.48)	988 (0, 4,999)	1.71 (0, 8.65)	−1.64 (−1.79, −1.50)
Caribbean	1,354 (564, 2,021)	5.45 (2.26, 8.12)	2,993 (1,167, 4,576)	5.54 (2.16, 8.45)	0.14 (0.11, 0.17)	252 (−59, 492)	1.0 (−0.23, 1.95)	428 (−95, 860)	0.79 (−0.18, 1.59)	−0.60 (−0.73, −0.48)	236 (0, 1,223)	0.94 (0, 4.83)	311 (0, 1,671)	0.58 (0, 3.1)	−1.48 (−1.55, −1.40)
Central Latin America	2,125 (845, 3,121)	2.75 (1.11, 4.05)	8,360 (2,900, 12,896)	3.39 (1.18, 5.23)	0.70 (0.60, 0.79)	505 (−116, 988)	0.66 (−0.15, 1.29)	907 (−210, 1,874)	0.37 (−0.09, 0.77)	−2.00 (−2.08, −1.91)	1,316 (0, 6,625)	1.7 (0, 8.53)	2,295 (0, 11,895)	0.93 (0, 4.83)	−2.18 (−2.26, −2.10)
Tropical Latin America	3,064 (1,144, 4,648)	3.64 (1.39, 5.52)	10,847 (3,358, 16,765)	4.27 (1.33, 6.6)	0.54 (0.46, 0.62)	1,515 (−344, 2,948)	1.7 (−0.39, 3.3)	2,971 (−675, 5,902)	1.15 (−0.26, 2.28)	−1.23 (−1.28, −1.19)	1,356 (0, 6,802)	1.58 (0, 7.95)	1,988 (0, 10,206)	0.78 (0, 4.01)	−2.36 (−2.42, −2.31)
North Africa and Middle East	5,381 (2,154, 8,061)	3.45 (1.4, 5.16)	13,716 (5,050, 21,143)	3.28 (1.22, 5.07)	0.04 (−0.10, 0.19)	745 (−148, 1,532)	0.47 (−0.09, 0.96)	1,051 (−209, 2,221)	0.25 (−0.05, 0.54)	−2.31 (−2.47, −2.15)	1,257 (0, 7,341)	0.75 (0, 4.4)	1,977 (0, 11,966)	0.45 (0, 2.75)	−1.58 (−1.64, −1.51)
South Asia	9,045 (5,038, 13,038)	1.64 (0.92, 2.36)	22,076 (10,679, 31,369)	1.55 (0.76, 2.19)	−0.27 (−0.35, −0.19)	5,716 (−1,372, 11,352)	1.01 (−0.24, 2)	11,825 (−2,570, 24,375)	0.81 (−0.18, 1.68)	−0.92 (−1.02, −0.82)	3,631 (0, 18,683)	0.62 (0, 3.21)	6,499 (0, 32,700)	0.45 (0, 2.25)	−0.95 (−1.03, −0.86)
East Asia	51,053 (23,740, 75,640)	6.46 (3.07, 9.56)	107,789 (38,058, 174,102)	5.15 (1.82, 8.30)	−0.80 (−0.86, −0.74)	45,020 (−9,645, 89,058)	5.36 (−1.14, 10.61)	9,457 (−1,822, 26,400)	0.47 (−0.09, 1.28)	−8.86 (−9.24, −8.49)	31,816 (0, 155,224)	3.77 (0, 18.42)	37,862 (0, 188,112)	1.76 (0, 8.69)	−2.54 (−2.74, −2.34)
Oceania	74 (37, 110)	2.89 (1.5, 4.17)	172 (87, 244)	2.53 (1.32, 3.57)	−0.39 (−0.47, −0.30)	14.73 (−3, 30)	0.55 (−0.11, 1.1)	32 (−7, 65)	0.47 (−0.1, 0.94)	−0.53 (−0.56, −0.51)	36 (0, 196)	1.36 (0, 7.12)	72 (0, 381)	1.06 (0, 5.52)	−0.83 (−0.89, −0.78)
Southeast Asia	12,856 (8,126, 16,936)	5.29 (3.37, 6.94)	38,667 (20,106, 50,505)	5.91 (3.28, 8.15)	0.30 (0.24, 0.37)	1,747 (−416, 3,427)	0.7 (−0.17, 1.37)	3,556 (−806, 7,101)	0.55 (−0.12, 1.09)	−0.88 (−0.93, −0.84)	2,264 (0, 11,385)	0.91 (0, 4.61)	3,572 (0, 18,178)	0.56 (0, 2.89)	−1.71 (−1.78, −1.65)
Central Sub-Saharan Africa	716 (413, 1,005)	3.61 (2.14, 5.02)	1,800 (987, 2,681)	3.69 (2.05, 5.61)	0.08 (−0.02, 0.18)	655 (−143, 1,321)	3.02 (−0.66, 5.98)	1,215 (−250, 2,449)	2.33 (−0.47, 4.62)	−1.00 (−1.10, −0.90)	142 (0, 838)	0.68 (0, 4.03)	262 (0, 1,544)	0.51 (0, 2.99)	−0.96 (−1.00, −0.92)
Eastern Sub-Saharan Africa	3,798 (2,195, 5,301)	5.44 (3.2, 7.57)	7,193 (3,756, 9,833)	4.93 (2.63, 6.71)	−0.51 (−0.61, −0.41)	2,717 (−630, 5,109)	3.76 (−0.88, 7.08)	4,766 (−946, 9,060)	2.96 (−0.59, 5.6)	−0.99 (−1.07, −0.91)	658 (0, 3,325)	0.9 (0, 4.5)	852 (0, 4,457)	0.54 (0, 2.79)	−1.90 (−1.99, −1.81)
Southern Sub-Saharan Africa	1,030 (556, 1,464)	4.12 (2.25, 5.87)	2,797 (1,466, 3,911)	5.24 (2.79, 7.33)	0.87 (0.60, 1.15)	772 (−177, 1,488)	2.88 (−0.66, 5.63)	1,582 (−347, 3,043)	2.8 (−0.61, 5.38)	−0.47 (−0.95, 0.00)	148 (0, 796)	0.55 (0, 3)	255 (0, 1,387)	0.45 (0, 2.48)	−0.69 (−1.01, −0.37)
Western Sub-Saharan Africa	2,081 (1,179, 2,851)	2.63 (1.52, 3.58)	4,581 (2,159, 6,430)	2.68 (1.31, 3.73)	0.18 (0.13, 0.23)	578 (−124, 1,141)	0.68 (−0.15, 1.35)	1,942 (−430, 3,930)	1.05 (−0.23, 2.13)	1.94 (1.73, 2.15)	479 (0, 2,559)	0.57 (0, 3.06)	880 (0, 4,609)	0.49 (0, 2.54)	−0.28 (−0.37, −0.20)

^*^Age-standardized mortality rates are calculated as per 100,000 population. Data were presented with 95% uncertainty intervals.

**Table 2:** DALYs and age-standardized DALY rates, and average annual percent changes (AAPCs) of gastrointestinal cancers attributable to dietary risks in 1990 and 2021*.

	Colon and rectum cancer					Esophageal cancer					Stomach cancer				
	1990		2021			1990		2021			1990		2021		
	DALYs	ASDR	DALYs	ASDR	AAPC of ASDR	DALYs	ASDR	DALYs	ASDR	AAPC of ASDR	DALYs	ASDR	DALYs	ASDR	AAPC of ASDR
Global	5810.3 (2146.0, 8636.2)	144.9 (53.1, 215.5)	9458.5 (3251.7, 14521.2)	109.7 (37.7, 168.5)	−0.88 (−0.90, −0.86)	2026.1 (−443.0, 4008.1)	49.0 (−10.7, 96.9)	1396.8 (−293.0, 2888.4)	16.0 (−3.4, 33.1)	−3.56 (−3.58, −3.53)	1845.6 (0, 9206.2)	44.5 (0, 222.3)	1804.6 (0, 8884.4)	20.8 (0, 102.4)	−2.45 (−2.47, −2.43)
Sex															
Male	2974.0 (1029.8, 4522.4)	159.1 (54.1, 241.1)	5302.7 (1686.7, 8243.1)	130.7 (41.6, 203.4)	−0.67 (−0.7, −0.65)	1452.0 (−319.3, 2893.3)	73.5 (−16.2, 146.7)	981.6 (−207.9, 2060.3)	23.7 (−5.1, 49.9)	−3.84 (−3.92, −3.76)	1221.1 (0, 6151.9)	62.2 (0, 314.7)	1231.3 (0, 6026.4)	29.9 (0, 146.7)	−2.42 (−2.52, −2.33)
Female	2836.3 (1095.5, 4187.8)	132.8 (51.1, 196.2)	4155.7 (1533.1, 6163.7)	91.0 (33.6, 134.7)	−1.36 (−1.41, −1.31)	574.1 (−110.4, 1164.0)	26.7 (−5.1, 54.0)	415.2 (−87.1, 844.9)	9.1 (−1.9, 18.6)	−3.32 (−3.40, −3.24)	624.6 (0, 3176.9)	28.7 (0, 146.2)	573.3 (0, 2941.0)	12.6 (0, 64.6)	−2.84 (−2.93, −2.74)
Region															
Central Asia	54.5 (16.1, 85.1)	109.9 (32.7, 171.5)	65.5 (17.7, 105.1)	75.6 (20.6, 121.2)	−0.98 (−1.08, −0.87)	27.2 (−5.9, 56.8)	56.5 (−12.2, 117.9)	5.6 (−1.0, 13.5)	6.9 (−1.3, 16.4)	−8.06 (−8.64, −7.49)	28.8 (0, 143.2)	58.0 (0, 288.4)	20.4 (0, 105.9)	23.1 (0, 119.4)	−2.80 (−2.88, −2.73)
Central Europe	289.2 (79.9, 450.0)	193.1 (53.5, 300.4)	412.5 (100.6, 651.2)	192.1 (46.3, 303.0)	−0.09 (−0.21, 0.04)	21.1 (−4.6, 43.4)	14.1 (−3.0, 28.9)	16.2 (−3.3, 34.0)	7.9 (−1.6, 16.7)	−2.38 (−2.57, 2.19)	55.1 (0, 277.9)	36.7 (0, 185.3)	34.9 (0, 170.9)	16.7 (0, 81.9)	−2.62 (−2.71, −2.54)
Eastern Europe	539.2 (125.2, 826.0)	191.5 (44.6, 293.5)	580.6 (144.5, 893.7)	167.8 (41.7, 257.9)	−0.76 (−0.91, −0.60)	60.7 (−13.0, 121.9)	21.3 (−4.6, 42.8)	47.9 (−9.4, 98.2)	14.1 (−2.8, 28.9)	−1.31 (−1.63, −0.98)	173.7 (0, 903.2)	61.6 (0, 320.7)	79.2 (0, 402.5)	23.5 (0, 119.4)	−3.40 (−3.52, −3.28)
Australasia	52.6 (12.5, 83.3)	227.7 (53.9, 360.3)	66.8 (15.7, 106.0)	131.3 (30.5, 207.9)	−1.99 (−2.08, −1.90)	4.9 (−1.1, 9.8)	21.1 (−4.8, 42.1)	8.5 (−1.9, 17.1)	16.6 (−3.7, 33.4)	−0.81 (−0.89, −0.73)	2.6 (0, 14.2)	11.3 (0, 61.4)	2.8 (0, 15.5)	5.7 (0, 31.1)	−2.16 (−2.26, −2.05)
High-Income Asia Pacific	334.0 (95.3, 513.8)	164.6 (47.4, 253.6)	555.4 (165.7, 866.4)	128.3 (36.5, 199.5)	−0.85 (−0.91, −0.79)	35.6 (−7.2, 74.1)	17.2 (−3.5, 35.9)	31.9 (−5.7, 72.8)	7.4 (−1.3, 16.8)	−2.56 (−2.72, −2.19)	151.9 (0, 743.7)	74.6 (0, 365.7)	99.0 (0, 495.3)	22.6 (0, 112.1)	−3.92 (−3.97, −3.87)
High-income North America	640.8 (139.4, 997.2)	186.5 (40.2, 290.3)	768.8 (149.0, 1206.4)	127.5 (24.3, 199.7)	−1.30 (−1.35, −1.25)	57.7 (−12.3, 115.4)	17.4 (−3.7, 34.8)	97.5 (−21.1, 196.1)	15.6 (−3.4, 31.4)	−0.25 (−0.40, −0.09)	31.6 (0, 165.4)	9.3 (0, 48.8)	32.6 (0, 166.5)	5.5 (0, 28.1)	−1.73 (−1.77, −1.68)
Southern Latin America	83.2 (25.1, 128.2)	180.1 (54.6, 277.3)	141.7 (39.1, 224.1)	165.2 (45.4, 261.5)	−0.01 (−0.14, 0.12)	18.2 (−4.3, 36.2)	39.3 (−9.2, 78.0)	16.0 (−3.7, 32.4)	18.5 (−4.3, 37.4)	−2.41 (−2.61, −2.22)	16.3 (0, 82.3)	35.0 (0, 176.9)	16.6 (0, 83.0)	19.4 (0, 97.0)	−1.73 (−1.77, −1.68)
Western Europe	1123.8 (251.7, 1747.9)	199 (44.2, 309.7)	1159.4 (241.5, 1811.5)	130.3 (26.6, 203)	−1.40 (−1.45, −1.35)	120.5 (−26.7, 240.9)	22.3 (−5.0, 44.6)	139.4 (−30.6, 282.2)	16.6 (−3.6, 33.6)	−0.97 (−1.08, −0.87)	127.5 (0, 667.8)	22.9 (0, 119.2)	76.2 (0, 392.3)	8.9 (0, 46.0)	−2.96 (−3.05, −2.88)
Andean Latin America	18.0 (8.8, 26.2)	84.7 (41.6, 122.6)	52.0 (20.3, 81.3)	88.8 (34.1, 135.4)	0.06 (−0.04, 0.16)	2.5 (−0.5, 4.9)	11.9 (−2.6, 23.5)	4.5 (−1.0, 8.9)	7.6 (−1.7, 15.0)	−1.54 (−1.66, −1.42)	13.4 (0, 68.1)	62.4 (0, 316.6)	23.1 (0, 116.7)	38.5 (0, 193.9)	−1.79 (−1.93, −1.64)
Caribbean	32.5 (13.7, 48.6)	123.9 (52, 185)	68.5 (27.1, 103.7)	127.6 (50.6, 193.1)	0.20 (0.16, 0.23)	6.3 (−1.5, 12.2)	23.9 (−5.5, 46.5)	10.9 (−2.4, 21.8)	20.1 (−4.4, 40.3)	−0.39 (−0.52, −0.27)	5.8 (0, 30.3)	21.9 (0, 114.6)	7.6 (0, 41.0)	14.2 (0, 76.7)	−1.31 (−1.41, −1.21)
Central Latin America	55.1 (21.3, 80.7)	62.4 (24.3, 91.6)	214.6 (72.2, 332.7)	83.8 (28.3, 130.0)	0.96 (0.87, 1.05)	12.5 (−2.9, 24.4)	14.7 (−3.4, 28.7)	21.4 (−5.0, 44.2)	8.4 (−2.0, 17.4)	−1.93 (−2.02, −1.84)	33.8 (0, 170.5)	38.6 (0, 194.2)	57.3 (0, 297.9)	22.4 (0, 116.5)	−2.00 (−2.09, −1.92)
Tropical Latin America	81.4 (29.9, 123.3)	84.5 (31.3, 128.3)	274.6 (83.7, 425.5)	105.3 (32.3, 163.1)	0.68 (0.59, 0.77)	42.5 (−9.7, 82.9)	43.5 (−9.9, 84.7)	78.6 (−17.9, 156.5)	29.8 (−6.8, 59.3)	−1.25 (−1.31, −1.19)	35.9 (0, 179.7)	37.3 (0, 186.6)	49.2 (0, 252.5)	18.9 (0, 96.8)	−2.33 (−2.39, −2.27)
North Africa and Middle East	154.0 (60.9, 232.3)	84.3 (33.6, 126.6)	370.0 (134.3, 570.9)	76.4 (27.9, 117.9)	−0.17 (−0.29, −0.04)	20.4 (−4.1, 42.7)	11.4 (−2.3, 23.6)	27.5 (−5.4, 57.2)	5.7 (−1.1, 12.0)	−2.62 (−2.81, −2.43)	37.0 (0, 214.6)	19.7 (0, 114.8)	54.8 (0, 325.7)	11.0 (0, 65.9)	−1.83 (−1.90, −1.77)
South Asia	271.5 (150.0, 390.5)	42.3 (23.5, 60.8)	610.9 (287.9, 877.5)	39.0 (18.6, 56.1)	−0.37 (−0.45, −0.29)	170.0 (−40.8, 336.8)	26.5 (−6.4, 52.5)	327.3 (−70.6, 675.4)	20.8 (−4.5, 43.0)	−1.00 (−1.10, −0.91)	112.6 (0, 573.8)	17.0 (0, 87.3)	180.8 (0, 908.1)	11.5 (0, 57.6)	−1.19 (−1.26,-1.11)
East Asia	1491.7 (689.0, 2203.4)	160.2 (74.6, 236.3)	2676.9 (928.0, 4313.1)	124.4 (43.3, 200.2)	−0.89 (−0.97, −0.80)	1239.1 (−269.4, 2453.5)	133.7 (−28.9, 264.6)	195.2 (−37.6, 546.7)	9.0 (−1.8, 25.3)	−9.81 (−10.22, −9.40)	910.2 (0, 4421.0)	96.6 (0, 469.1)	906.4 (0, 4574.2)	41.1 (0, 206.6)	−2.88 (−3.06, −2.70)
Oceania	2.3 (1.1, 3.4)	69.8 (35.7, 102.7)	5.2 (2.6,)	61.6 (31.2, 87.1)	−0.38 (−0.45, −0.31)	0.4 (−0.08, 0.9)	13.5 (−2.6, 27.5)	0.9 (−0.02, 1.9)	11.3 (−2.3, 22.9)	−0.56 (−0.59, −0.53)	1.0 (0, 5.9)	32.7 (0, 178.4)	2.1 (0, 11.6)	25.5 (0, 135.0)	−0.82 (−0.89, −0.76)
Southeast Asia	375.1 (236.5, 496.5)	133.6 (84.3, 176.2)	990.5 (537.0, 1363.5)	143.6 (78.1, 197.2)	0.17 (0.11, 0.23)	50.4 (−11.9, 98.9)	18.1 (−4.3, 35.5)	97.6 (−22.1, 197.0)	13.8 (−3.1, 27.8)	−0.97 (−1.01, −0.93)	66.7 (0, 336.5)	23.5 (0, 118.1)	97.7 (0, 498.0)	14.0 (0, 71.3)	−1.85 (−1.91, −1.78)
Central Sub-Saharan Africa	20.8 (11.7, 29.5)	86.3 (49.9, 120.8)	52.7 (28.4, 79.0)	87.0 (47.9, 129.9)	0.03 (−0.07, 0.13)	19.3 (−4.2, 39.1)	77.3 (−16.8, 156.0)	35.8 (−7.4, 72.3)	57.9 (−11.9, 116.5)	−1.12 (−1.22, −1.02)	4.2 (0, 25.0)	17.0 (0, 100)	7.9 (0, 46.2)	12.5 (0, 73.9)	−1.02 (−1.06, −0.98)
Eastern Sub-Saharan Africa	108.3 (62.4, 152.3)	134.2 (77.6, 187.9)	195.6 (98.6, 268.9)	109.1 (56.7, 149.0)	−0.91 (−1.02, −0.80)	78.4 (−18.0, 147.7)	96.4 (−22.3, 181.3)	136.2 (−26.7, 258.9)	73.3 (−14.5, 139.4)	−1.13 (−1.22, −1.03)	19.7 (0, 99.8)	23.4 (0, 118.1)	24.5 (0, 129.5)	12.9 (0, 67.7)	−2.21 (−2.32, −2.11)
Southern Sub-Saharan Africa	27.7 (14.7–39.4)	95.7 (51.2, 136.6)	75.3 (38.5, 105.8)	123.0 (63.7, 172.4)	0.99 (0.70, 1.28)	22.7 (−5.2, 43.7)	76.8 (−17.5, 147.6)	44.5 (−9.8, 85.6)	71.2 (−15.7, 137.6)	−0.65 (−1.12, −0.17)	4.4 (0, 23.7)	14.0 (0, 75.1)	7.3 (0, 39.1)	11.3 (0, 59.3)	−0.75 (−1.08, −0.43)
Western Sub-Saharan Africa	54.4 (30.1–75.2)	60.0 (33.6, 82.5)	121.0 (54.7–172.3)	58.7 (27.4, 82.7)	0.02 (−0.03, 0.07)	15.7 (−3.4, 31.4)	16.9 (−3.7, 33.7)	53.4 (−11.9, 107.9)	25.1 (−5.6, 50.8)	1.80 (1.61, 2.00)	13.2 (0, 70.7)	14.6 (0, 78.1)	24.1 (0, 126.9)	11.5 (0, 62.2)	−0.48 (−0.55, −0.40)

^*^DALYs are represented with the unit of 1,000. Age-standardized DALY rates are calculated as per 100,000 population. Data were presented with 95% uncertainty intervals.

### GI cancer burden attributable to dietary risks stratified by sex, age and geographical location

**Figure 2:**
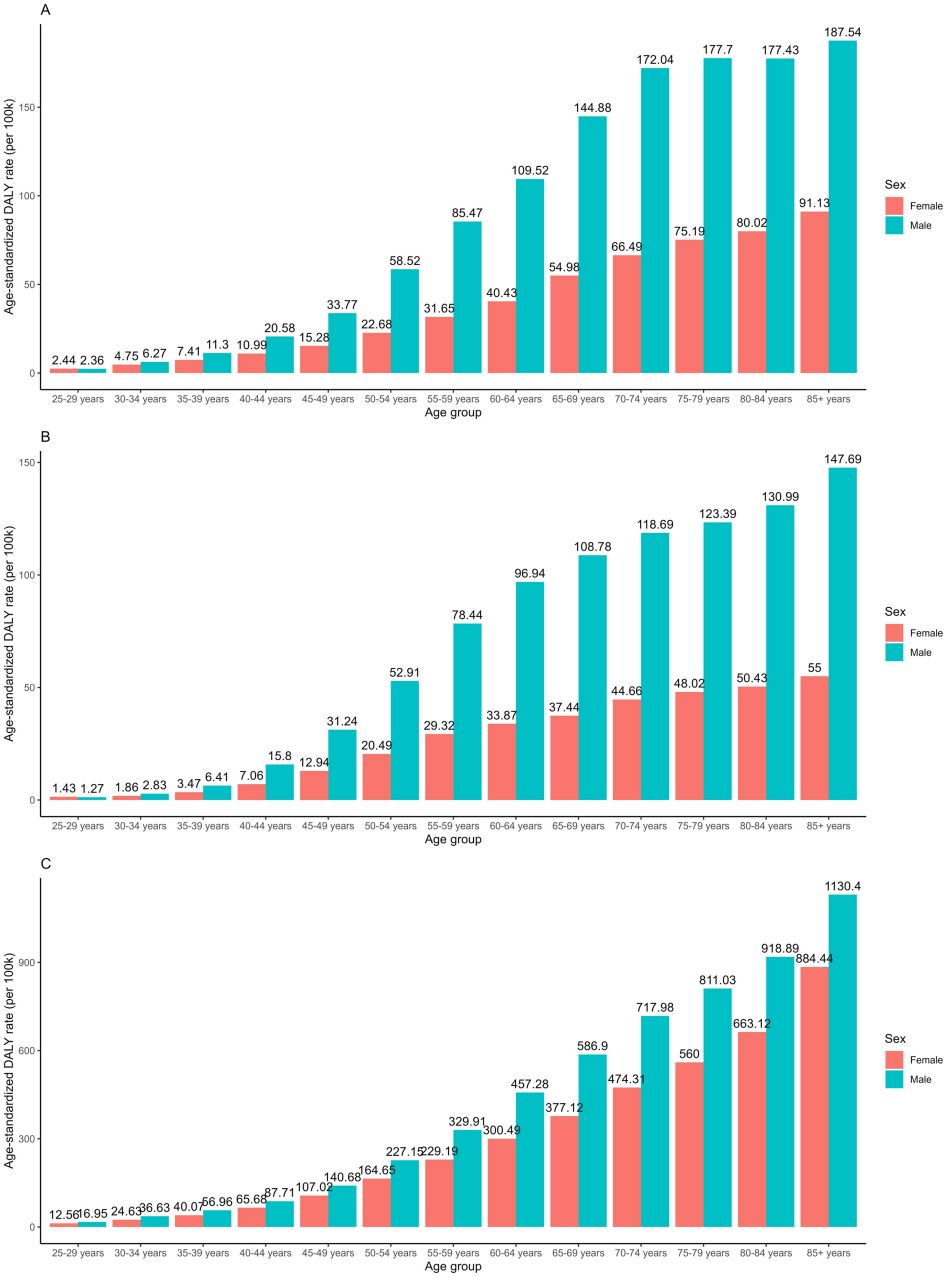
Age-specific DALY rates of **(A)** stomach cancer; **(B)** esophageal cancer and **(C)** colorectal cancer attributable to dietary risks between sex in 2021.

Globally, males consistently bear a significantly higher burden of all GI cancers attributable to dietary risks, marked by both higher global ASRs and lower decreasing temporal trends. The only exception was the AAPCs in the ASDR of esophageal cancer, where males exhibited an annual declining rate of −4.14% compared to −3.92% in females. Diet-attributed CRC showed the greatest disparity with the temporal trends between 1990 and 2021. The AAPC of the ASMR and ASDR were −0.65% and −0.67% in males, compared to −1.30% and −1.36% in females, a two-fold difference. The sex disparity was consistent while stratified by age groups, as consistently higher age-specific rates were observed among males compared to females (Figure 2). In addition, the sex-specific rate increases in both males and females as age increases, with the highest DALY rates reported in the 85 + age group.

**Figure 3:**
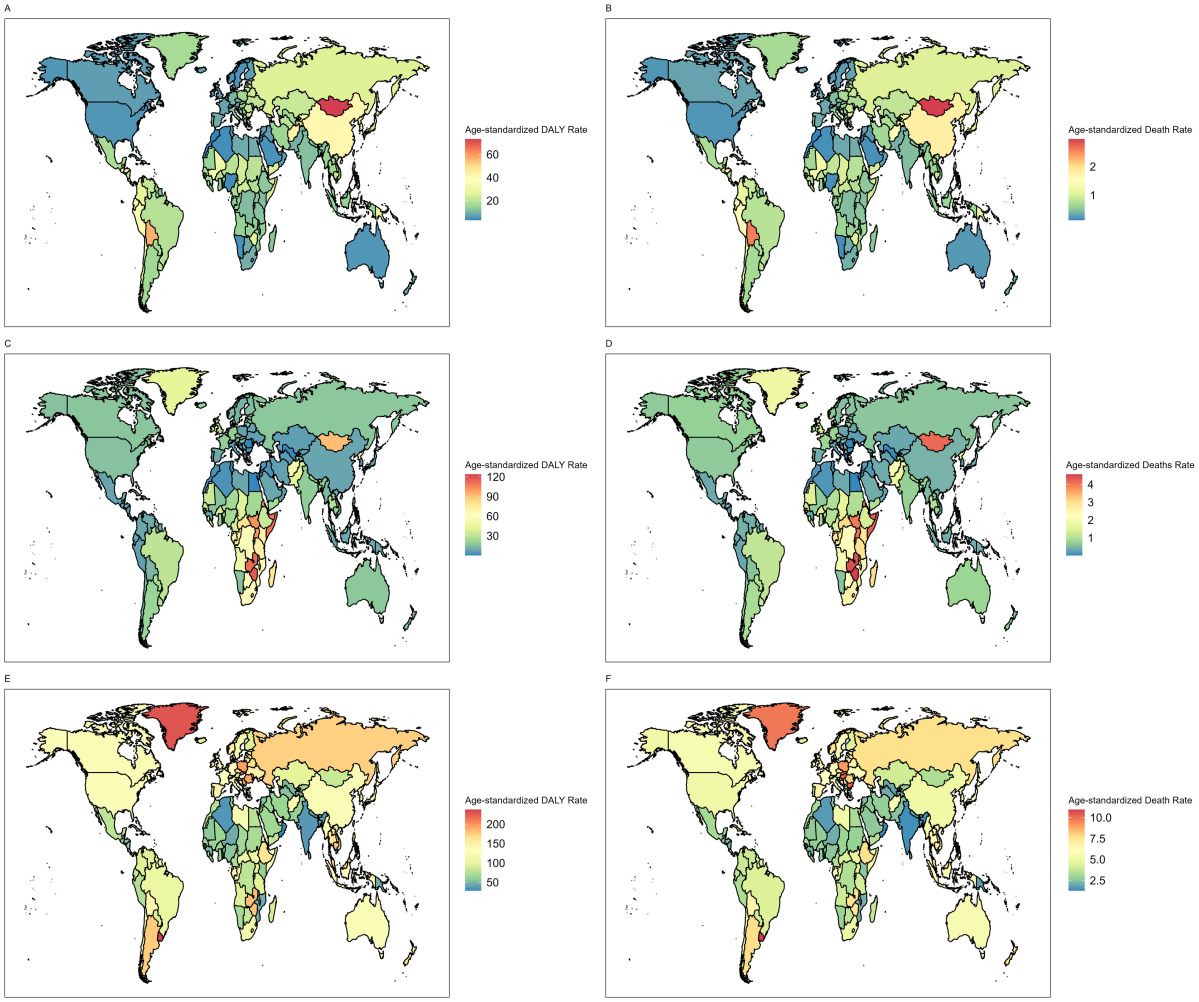
Age-standardized DALY rates of **(A)** stomach cancer **(C)** esophageal cancer **(E)** colorectal cancer attributable to dietary risks in 2021. Age-standardized death rates of **(B)** stomach cancer, **(D)** esophageal cancer and **(F)** colorectal cancer attributable to dietary risks in 2021.

The global distribution of GI cancer burden attributable to dietary risks varied substantially across cancer types and regions (Figure 3). In 2021, East Asia had the highest burden of stomach cancer (ASMR: 1.76 per 100,000; ASDR: 41.09 per 100,000), followed by Andean Latin America and Oceania, while high-income regions such as North America, Australasia, and Western Europe reported the lowest rates. Esophageal cancer showed its greatest burden in Sub-Saharan Africa, where ASDRs exceeded 50 per 100,000 and ASMRs surpassed 2 per 100,000, whereas the lowest burdens were observed in North Africa and the Middle East, Central Asia, and high-income Asia Pacific. For CRC, higher burdens were concentrated in higher-SDI regions such as Central and Eastern Europe and Southern Latin America, while the lowest ASRs were seen in low-SDI regions including South Asia, Western Sub-Saharan Africa, and Oceania.

**Figure 4:**
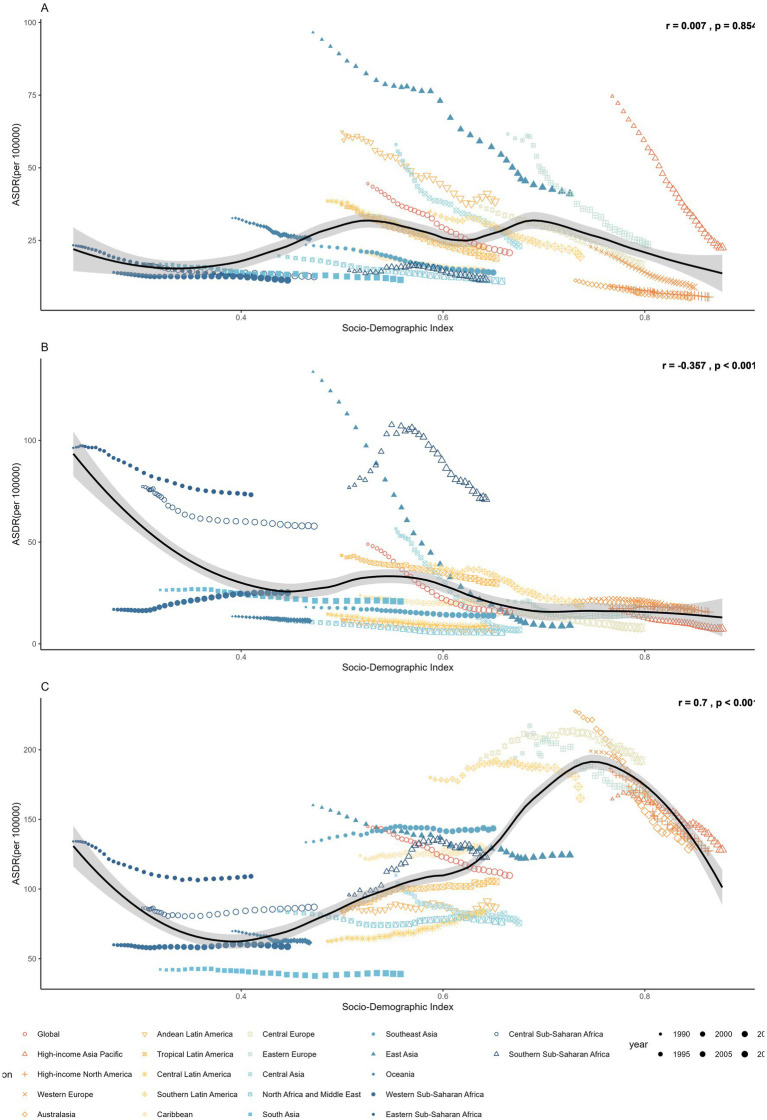
The ASDR of **(A)** stomach cancer, **(B)** esophageal cancer and **(C)** colorectal cancer attributable to dietary risks across 21 regions, 1990–2021.

**Figure 5:**
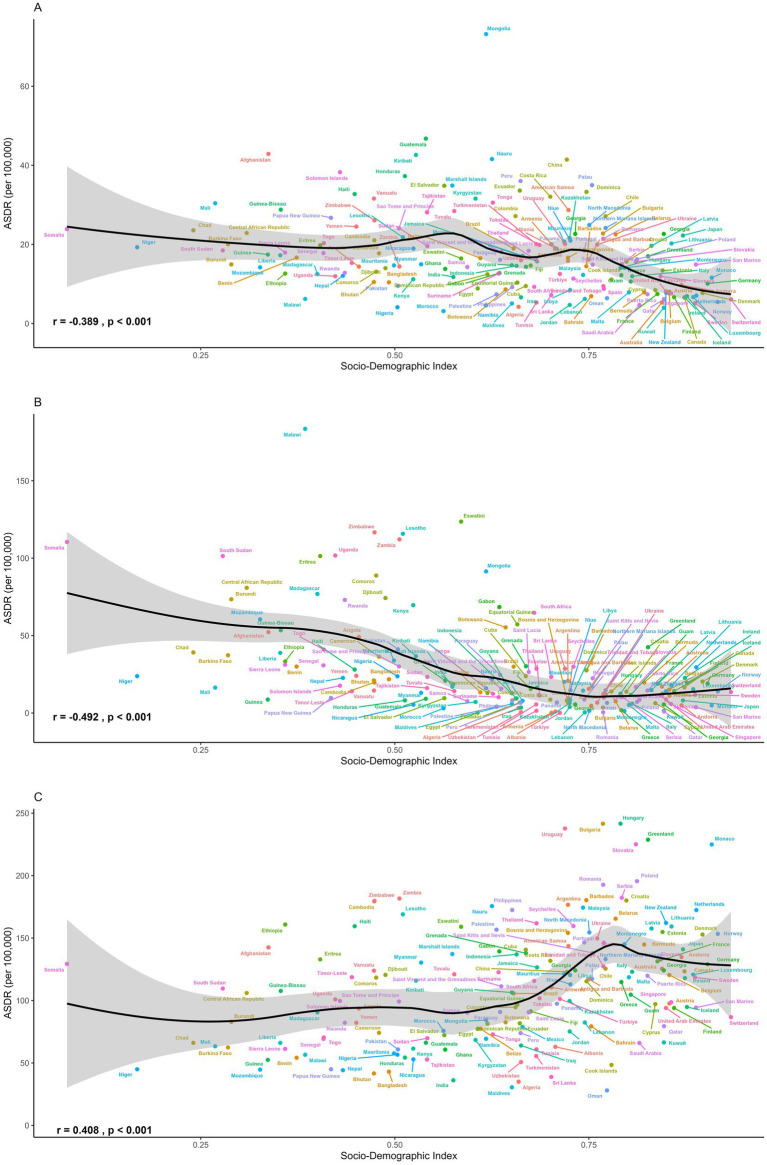
The ASDR of **(A)** stomach cancer, **(B)** esophageal cancer and **(C)** colorectal cancer attributable to dietary risks across 204 countries and territories in 2021.

Regional disparities also persisted in temporal trends (Figure 4 and Tables 1, 2). Although stomach cancer attributable to dietary risks declined consistently across all 21 regions, high-SDI and high–middle-SDI regions experienced the most pronounced reductions, exceeding 3% annually in areas such as high-income Asia Pacific, Eastern Europe, and Western Europe (Figure 4A). Country-level analyses further revealed a negative association between SDI and stomach cancer burden (Figure 5A), a pattern also evident for esophageal cancer (Figure 5B). Regionally, East Asia and Central Asia showed the steepest declines in esophageal cancer, while Western Sub-Saharan Africa was the only region with rising rates (AAPC: 1.94% for ASMR; 1.80% for ASDR) (Figure 4B). For CRC, high-SDI regions showed the largest decreases, outpacing global averages, whereas low-SDI and low-middle-SDI regions exhibited increasing trends (Figure 4C). At the global level, analysis of 204 countries and territories revealed a positive association between SDI and diet-attributed CRC burden, as ASDR increased with SDI but plateaued when SDI exceeded 0.75 (Figure 5C).

### CRC burden attributable to specific dietary risks (level 3 risks)

**Figure 6:**
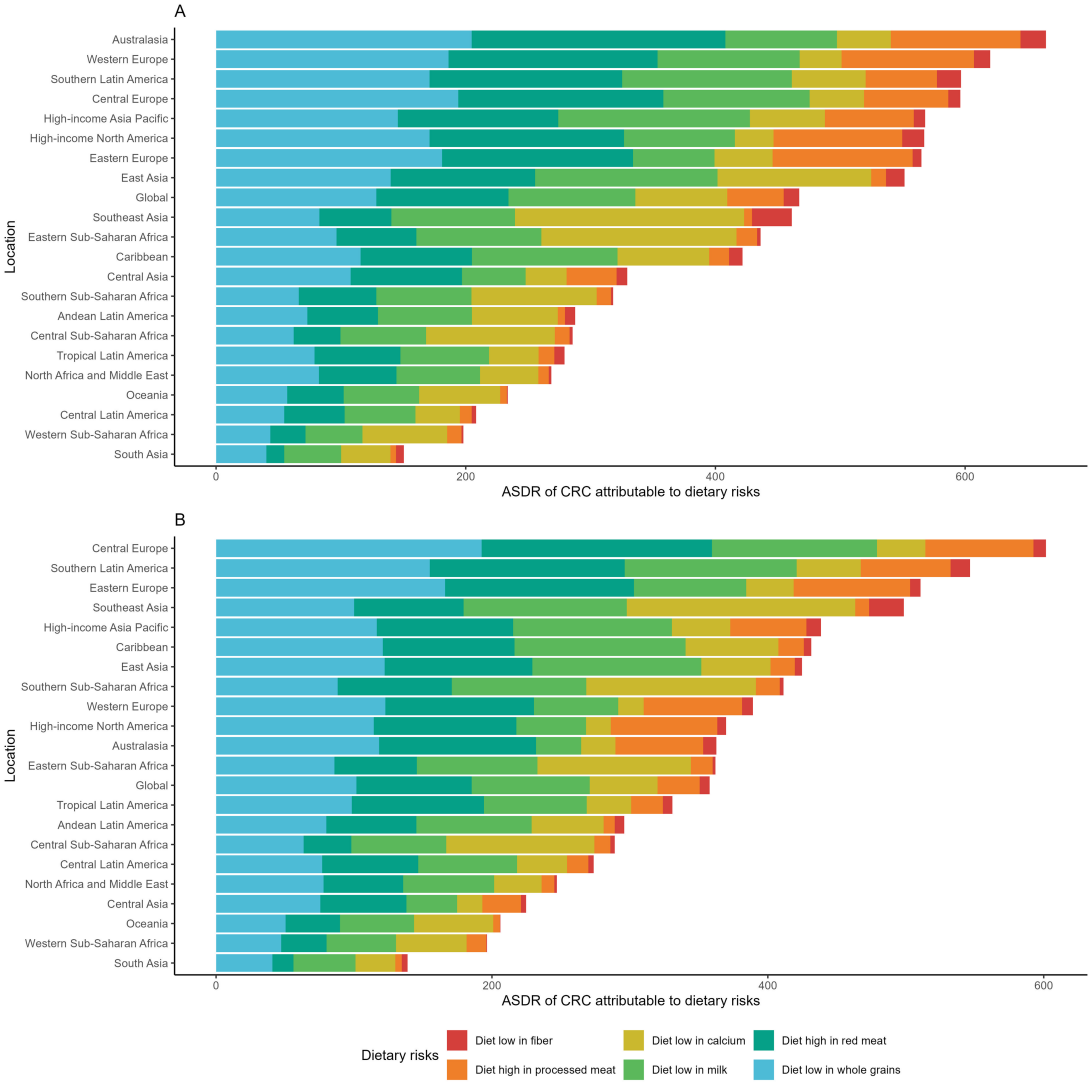
**(A)** Age-standardized DALY rates of colorectal cancer attributable to dietary risks across GBD regions in 1990. **(B)** Age-standardized DALY rates of colorectal cancer attributable to dietary risks across GBD regions in 2021.

GBD identified six dietary risks contributing to CRC. We compared the ASDRs of CRC attributable to these risks globally and across 21 regions in 1990 and 2021 (Figures 6A,B). In both years, diets low in whole grains, low in milk, and high in red meat were consistently the three leading dietary risks. In 2021, the ASDRs of CRC attributable to these factors were 50.19, 42.99, and 41.99 per 100,000 population, respectively. Substantial regional disparities were observed in the risk-specific burden. Central and Eastern Europe, Southern Latin America, and several high-income regions bore the greatest burden of CRC attributable to low whole-grain intake and high red meat consumption. These regions also exhibited elevated ASDRs linked to high processed meat intake, with several surpassing 30 per 100,000 population. In contrast, diets low in milk and calcium had stronger impacts in parts of Asia and Africa, including Southeast Asia and Sub-Saharan Africa regions. Among all dietary risks, low fiber intake contributed the smallest burden overall. However, Southeast Asia was disproportionately affected, with an ASDR of 12.40 per 100,000 population in 2021.

### Projection and forecasts of GI cancer burden attributable to dietary risks in 2040

**Figure 7:**
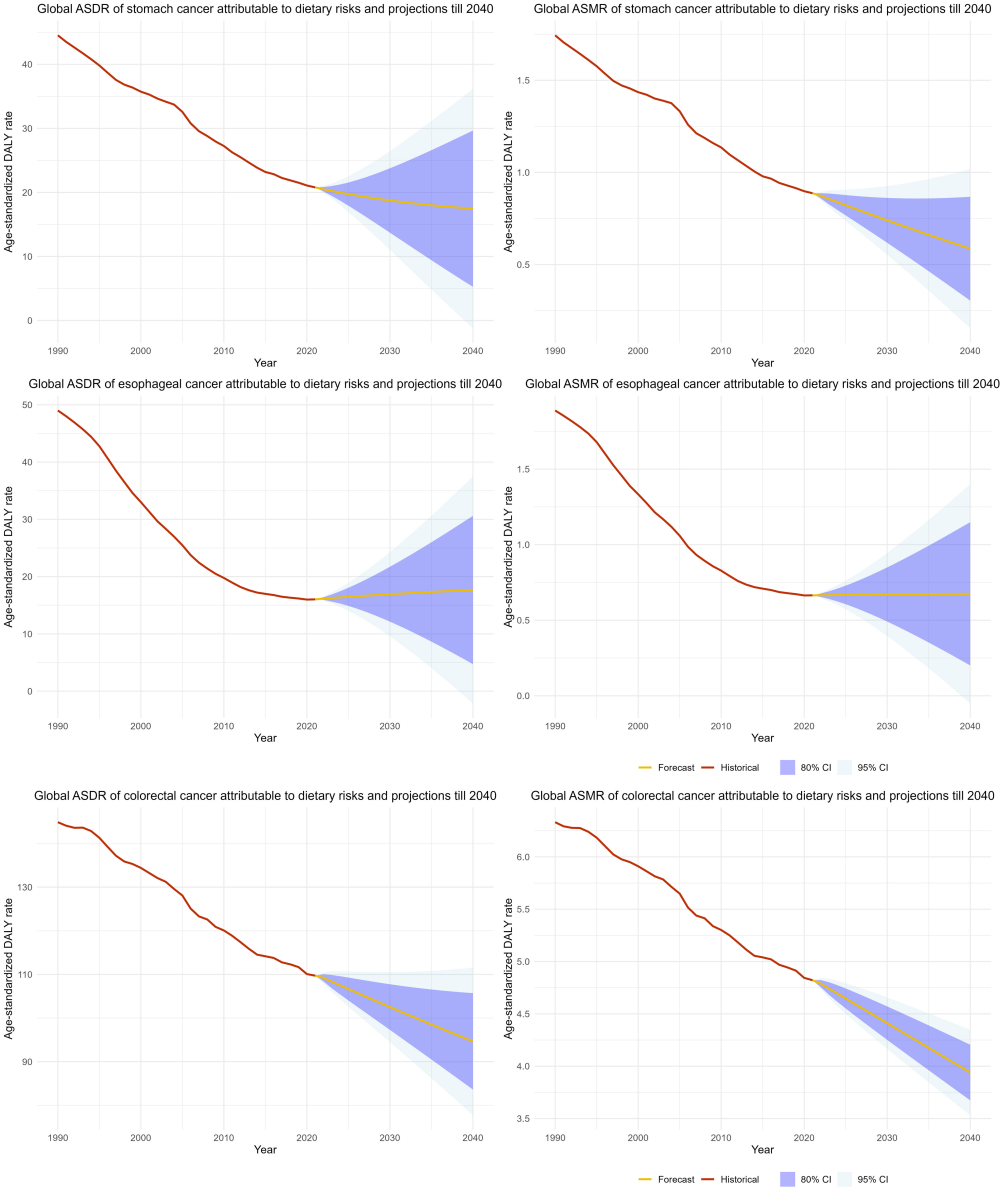
The projection and forecasted age-standardized mortality and age-standardized DALY rate of GI cancers attributable to dietary risks till 2040.

We projected the disease burden of GI cancers attributable to dietary risks through 2040 using ARIMA models (Figure 7). The ASRs of stomach cancer and colorectal cancer (CRC) are expected to decline steadily, whereas the ASRs of esophageal cancer are projected to remain largely stable over the next two decades. By 2040, the global ASMR and ASDR for stomach cancer are projected to decrease to 0.59 (95% CI: 0.15–1.02) and 17.46 (95% CI: 0.03–35.11) per 100,000 population, respectively. CRC is estimated to reach an ASMR of 3.94 (95% CI: 3.53–4.35) and an ASDR of 94.66 (95% CI, 77.77–111.56) per 100,000 population. In contrast, the projected burden of esophageal cancer shows no significant change, with global ASMR and ASDR of 0.67 and 17.67 per 100,000 population in 2040.

## Discussion

Our study systematically investigated the burden, trends and disparities of GI cancers attributable to dietary risks over the past three decades. Deaths and DALYs resulting from diet-attributable CRC nearly doubled on the global scale between 1990 and 2021. During this period, a decrease in deaths and DALYs associated with esophageal cancer was recorded, while the disease burden of stomach cancer associated with a high-sodium diet exhibited minimal changes. Despite these fluctuations, numerous low SDI and lower-middle SDI countries continued to experience substantial increases in GI cancers attributable to dietary risks. A declining trend in global ASMRs and ASDRs was evident for all three GI cancers linked to dietary risks. Regions with high SDI and high-middle SDI consistently displayed significant decreases, whereas low SDI and lower-middle SDI regions demonstrated relatively minor changes or even increasing trends. Noteworthy is the approximately 2% annual increase in age-standardized rates of esophageal cancers attributable to diet low in vegetables in Western Sub-Saharan Africa, the sole region showing an increase.

Our findings are broadly consistent with previous GBD analyses that have documented evolving yet uneven trends in gastrointestinal cancers worldwide. Earlier GBD studies (GBD 2017 and GBD 2019) similarly reported a global rise in colorectal cancer incidence and mortality, contrasted by steady declines in stomach and esophageal cancers (19–21). The present analysis based on GBD 2021 data reaffirms these trajectories while highlighting widening disparities across socioeconomic regions. Consistent with prior evidence, high-SDI regions have achieved marked reductions in age-standardized mortality and DALYs through advances in screening, early detection, and treatment, whereas low- and middle-SDI regions continue to experience rising burdens linked to rapid dietary transitions and limited healthcare infrastructure (19–21). The persistence of stomach cancer burden and the expanding impact of colorectal cancer in developing regions parallel the trends described in recent GBD updates, underscoring that uneven progress in dietary improvement and cancer control continues to shape the global landscape of GI cancers.

Our study findings align with prior research, revealing a higher incidence of deaths and DALYs attributed to diet-related GI cancers in males compared to females (22–24). In 2021, the global ASRs for esophageal and stomach cancers linked to dietary factors were approximately 2.5 times higher in males than in females, while the ASRs for CRC attributable to dietary risks were 40% higher in males compared to females. The prevalence of GI cancer burden in males is likely influenced by a combination of biological, behavioral, and environmental factors. Endogenous estrogen has been shown to exert protective effects, particularly against CRC, by modulating inflammatory pathways, influencing bile acid metabolism, and regulating DNA repair mechanisms (25, 26). Estrogen may also reduce oxidative stress and limit colonic epithelial proliferation, thereby lowering carcinogenesis risk (27, 28). The Women’s Health Initiative study was a double-blinded, placebo controlled randomized controlled study to investigate the role of estrogen plus progestin with colorectal cancer risk. Data from 16,608 postmenopausal women showed an decreased risk of CRC among women receiving estrogen with extended follow-ups (29, 30). In stomach cancer, estrogen has been hypothesized to inhibit *Helicobacter pylori*-induced inflammation, contributing to the lower incidence observed in females (31). Behavioral and lifestyle factors further amplify these disparities. Men have higher rates of alcohol consumption and tobacco use, both established carcinogens for esophageal and stomach cancers (32–34). In addition, higher levels of visceral adiposity and central obesity in men promote insulin resistance, chronic inflammation, and altered adipokine signaling, which increase CRC risk (35–37). The less pronounced sex disparity in CRC may be attributed to a more even distribution of the dietary and lifestyle risk factors (e.g., diet, sedentary lifestyles, etc.) along with the widespread implementation of CRC screening programs among all genders (38). However, further research is essential to elucidate sex-specific mechanisms to enhance prevention and treatment strategies effectively.

With respect to the age-specific burden of GI cancers attributable to dietary risks, we observed a progressive increase in incidence with advancing age, peaking among individuals aged 85 years and older. Sharp rises in age-specific rates were consistently evident beginning in the 40–44 and 45–49 age groups across all three GI cancers. While individuals over 50 years old bear a significantly higher burden of GI cancers, the incidence rates have shown a downward trend in recent decades, which is likely due to the progress with enhanced screening practices, early diagnosis and treatment advances (39, 40). On the other hand, GI cancers occurring before the age of 50, which refers to early-onset GI cancers, have shown steadily increasing incidence rates, particularly among high-income countries (41, 42). The rising incidence of early-onset CRC has been reported since the middle 1990s in the USA, with age-adjusted rates increasing from 5.9 to 8.4 per 100,000 population between 2000 and 2017 (43). Similarly, data from regional and national cancer databases revealed rising CRC incidence among several European countries, with consistent and significantly positive annual increasing rates across different countries and age groups (44, 45). Although early-onset CRC has received considerable attention, early-onset cancer diagnoses in other GI tract locations have also been investigated and reported (41). Data from the US Surveillance, Epidemiology and End Results (SEER) database indicated rising incidence rates of pancreatic cancer, esophageal cancer and introhepatic cholangiocarcinoma occurring among individuals between the age of 20 to 49 (46–48). Importantly, while improvements in diagnostic and screening practices may partially account for increased case detection, these changes are insufficient to explain the consistent upward trajectory across diverse digestive cancer sites. The drivers of early-onset GI cancers are multifactorial, with behavioral and environmental exposures playing crucial roles (41, 42). Previous research indicated Westernized diets, obesity and sedentary behaviors as main risk factors for early-onset GI cancers (49–51). Westernized dietary patterns characterized by high intake of red meat, sodium and saturated fats, with limited fruit, vegetable and whole grain consumption are well-established risk factors for various GI cancers. Studies have shown that Westernized diets elevate CRC risk by altering the gut microbiome to favor pro-carcinogenic species that generate genotoxic secondary bile acids and reduce protective short-chain fatty acids (52, 53). These dietary components directly promote DNA damage and oncogenic signaling through chronic mucosal inflammation and cellular proliferation. Studies have investigated other dietary components with the association of GI cancer risks. Data from the Nurse’s Health Study suggested that increased sugar-sweetened beverage intake, as well as reduced levels of vitamin D levels were associated with a higher risk of early-onset CRC (54, 55). These findings highlight the urgent need to adapt screening strategies in parallel with the shifting epidemiologic patterns. Given the robust evidence supporting the efficacy of screening and the escalating burden of early-onset GI cancers, it is recommended to streamline screening recommendations for younger adults (56). The American Cancer Society now advocates for CRC screening from the age of 45 onwards (57). Additionally, targeting individuals with elevated risks such as high BMI, a family history of GI cancers, or other GI tract conditions could enhance early detection efforts and alleviate the burden among younger adults (58, 59). Furthermore, expanding tailored screening beyond CRC, particularly in populations experiencing disproportionate increases in early-onset GI cancers, may be critical for mitigating the growing burden among younger adults.

Significant disparities in the burden of gastrointestinal cancers attributable to dietary risks closely mirror the SDI, with low and low-middle SDI countries bearing a disproportionately high burden of esophageal cancers and stomach cancers. This pattern is likely due to the complex interplay of economic constraints and culturally ingrained food practices that are characteristic of many low-resource settings. Structural inequities, such as limited access to high-quality and fresh foods, as well as inadequate healthcare resources, contribute to the elevated burdens in low SDI countries (60, 61). Diet-attributed stomach cancer displayed the highest age-standardized rates in East Asia and Andean Latin America, aligning with findings from previous epidemiological studies (62). In East Asia, the dietary habits are historically high in salted, pickled, and preserved foods, which are merely cultural preferences but also practical, low-cost solutions for food preservation in the absence of widespread refrigeration (63). Excess sodium directly damages the gastric mucosa and enhances the pathogenicity of *Helicobacter pylori*, a well-established carcinogen strongly associated with gastric cancer (64, 65). The extremely high prevalence of chronic *H. pylori* infection in East Asian populations (often exceeding 50%) amplifies these dietary effects and helps explain the region’s persistently elevated stomach cancer rates (66). By contrast, the highest global burden of esophageal cancer attributable to dietary risks was observed in Sub-Saharan Africa. Insufficient vegetable intake, a major dietary risk factor, is widespread in this region due to limited food diversity, economic barriers, and reliance on starchy staples (67). Low intake of antioxidant and micronutrient-rich foods (e.g., green leafy vegetables, fruits, legumes) reduces protection against oxidative stress and DNA damage in esophageal tissues, increasing susceptibility to carcinogenesis. Other region-specific exposures, such as micronutrient deficiencies (zinc, selenium), frequent consumption of very hot beverages, alcohol use, and exposure to dietary carcinogens such as polycyclic aromatic hydrocarbons from poorly ventilated cooking methods, may further compound the impact of inadequate vegetable intake (68, 69). To address these burdens and achieve health equity requires interventions that move beyond public health messaging to include economic development, food system improvements, and culturally sensitive strategies that make healthier choices accessible and viable.

Colorectal cancers, the third leading cause of cancer mortality worldwide, remain most prevalent in high-SDI countries (58). Previous studies have consistently reported positive associations of CRC cancer burden with SDI, which is largely driven by lifestyle, demographic and environmental factors (20). Beyond established risks such as Westernized dietary patterns, obesity, hyperglycemia, and sedentary behaviors, emerging evidence suggests that early-life exposures, such as antibiotic use, may disrupt the gut microbiome and increase CRC susceptibility later in life (70). However, despite the heightened burden, high SDI counties and regions showed the greatest decline of CRC burden, which is likely due to improved diagnosis, treatment and public awareness among these locations (60). Our findings reflect these broader epidemiological transitions. Historically concentrated in high SDI regions, Western dietary and lifestyle patterns are now increasingly observed in middle and low SDI countries, contributing to the rising CRC burden in these settings. Our study indicated that diets high in red meat and diets low in milk and whole grains are the major dietary risks for the burden of CRC, with Europe, Southern Latin America and Southeast Asia regions having the highest risk-specific age-standardized rates. A previous large-scale meta-analysis concluded that CRC risk decreases 13% for each 400 g/day increase of dairy products intake. There are several mechanisms to explain why these nutrients are associated with CRC risk (71). For example, whole grains are rich in dietary fiber, antioxidants and phytochemicals, which play crucial roles in maintaining gut health, regulating insulin levels, and reducing inflammation in the colon, collectively lowering the risk of CRC (72, 73). As CRC has been associated with multiple risk factors, it is essential to understand the dietary patterns and risk of CRC. A systematic review study conducted by the Global Cancer Update Programme (CUP Global) indicated strong-probable evidence with increased CRC risk with empirical dietary index for hyperinsulinemia (EDIH) and empirical dietary inflammatory patterns (EDIP) (74). Similarly, data from the Health Professional Follow-up study and Nurses’ Health Study suggested lowered CRC risk with prudent dietary patterns, which showed consistent effects regardless of anatomic or molecular subtype (75). The significant deviation among different dietary risk factors of CRC across various regions suggests target-specific and content-specific dietary public health policy and interventions to be implemented in the future. An overall healthy dietary pattern that promotes whole-grain and dairy consumption, reduces red and processed meat intake, and encourages microbiome-supportive dietary patterns may be critical to alleviating the global burden of CRC.

Content-specific and evidence-based strategies are imperative to translate these findings into effective public health actions. For dietary interventions, public health policies should actively promote the adoption of guidelines established by the World Health Organization (WHO) and the World Cancer Research Fund (WCRF), which recommend limiting red and processed meat intake to under 500 g per week, reducing sodium consumption to less than 2 g/day, and increasing the daily intake of dietary fiber, fruits, vegetables, and whole grains (76, 77). These guidelines should be operationalized through fiscal policies (e.g., sugar-sweetened beverage taxes, subsidies for fresh foods), front-of-package nutrition labelling and restrictions on marketing unhealthy foods. Concurrently, public health policy should prioritize the strategic application of innovative, non-invasive screening technologies, coupled with enhanced, risk-stratified screening protocols. For colorectal cancer, the adoption of sensitive, non-invasive tests like fecal immunochemical tests (FIT) and multi-target stool DNA (mt-sDNA) tests can significantly boost participation in screening programs, particularly among younger cohorts and in resource-limited settings where colonoscopy capacity is constrained (78, 79). Furthermore, screening must be proactively targeted to high-risk individuals beyond age alone. This includes implementing early and more intensive surveillance for those with a family history of GI cancers, genetic predispositions (such as Lynch syndrome), personal history of conditions like inflammatory bowel disease, or those with metabolic risk factors like high BMI (80, 81). Collectively, these multifaceted strategies hold the potential to substantially reduce the global burden of GI cancers by addressing modifiable dietary risks and improving early detection across diverse populations.

There were several limitations with our study. The major limitation was the non-availability of data from cancer registries in certain countries and regions, particularly in low and low-middle income countries. Relying on alternative data sources like vital registration and verbal autopsy due to the absence of population-wide cancer registries likely led to an underestimation of the disease burden. Moreover, our study could not fully address various confounding factors, such as genetic predispositions and environmental risk exposures, potentially introducing bias to the estimates of disease burden patterns. Furthermore, our study was unable to estimate the attributable burden based on the anatomical or histological subtypes of GI cancers, such as the cardia and non-cardia subtypes of stomach cancer, along with the esophageal squamous cell carcinoma (SCC) and esophageal adenocarcinoma (OAC) subtypes of esophageal cancer, which might show distinct disease burden across different subtypes. Last but not least, limitations were present in the measurement of dietary exposures, including unaccounted dietary risk factors like the consumption of ultra-processed foods, which are strongly linked to various cancer phenotypes (82, 83). It is important to note that no singular dietary pattern or score can comprehensively capture a healthy diet. The evolving field of dietary pattern research has increasingly focused on sustainable healthy diets, which intertwine with biodiversity, climate change, and environmental health concerns (84).

In conclusion, our study conducted a comprehensive analysis of the global burden of gastrointestinal cancers attributed to dietary risks using the latest publicly available GBD 2021 database. Of the three GI cancers studied, colon and rectum cancer contributed the leading cause of mortality and DALYs worldwide. Despite observed declines in age-standardized rates, distinct patterns and trends of GI cancers are evident across diverse geographical regions, with diet-attributed GI cancers are still significant public health challenges in low SDI and lower-middle SDI countries. Ensuring access to fresh and nutritious foods, enhancing screening practices, and improving healthcare availability are crucial strategies for low SDI and lower-middle SDI countries to mitigate the burden of GI cancers. Conversely, lifestyle adjustments and dietary modifications are imperative for higher SDI countries. Addressing these issues will be vital in tackling the burden of GI cancers and advancing public health outcomes globally.

## Data Availability

The original contributions presented in the study are included in the article/supplementary material, further inquiries can be directed to the corresponding author.

## Footnotes

^1^ https://vizhub.healthdata.org/gbd-results/
